# Editorial: Temporal Dynamics of Reward Processing in Humans: From Anticipation to Consummation

**DOI:** 10.3389/fpsyg.2020.01901

**Published:** 2020-08-19

**Authors:** Ya Zheng, Ruolei Gu, Daniela M. Pfabigan

**Affiliations:** ^1^Department of Psychology, Dalian Medical University, Dalian, China; ^2^Key Laboratory of Behavioral Science, Institute of Psychology, Chinese Academy of Sciences, Beijing, China; ^3^Department of Psychology, University of Chinese Academy of Sciences, Beijing, China; ^4^Department of Behavioural Medicine, Faculty of Medicine, University of Oslo, Oslo, Norway

**Keywords:** reward, anticipation, consummation, temporal dynamics, individual differences

People often want what they like, and like what they want. However, this lay knowledge is inconsistent with findings in reward-related disorders (Treadway and Zald, 2011; Whitton et al., 2015; Nusslock and Alloy, 2017). For example, patients suffering from depression and schizophrenia sometimes show intact hedonic responses to pleasurable stimuli, but are less willing to expend effort to acquire rewards (Culbreth et al., 2018). Drug addiction is characterized by an excessive craving for drugs, but is rarely companied by the expected positive hedonic responses (Robinson and Berridge, 1993). Individuals with anorexia-type eating disorders have normal levels of “wanting,” but reduced levels of “liking” of foods (Berridge et al., 2010). These clinical observations indicate that reward processing is not a homogenous construct but consists of two mainly successive phases, anticipation and consummation, as it unfolds over time (Rangel et al., 2008; Romer Thomsen et al., 2015). Although the dissociation between reward anticipation and consummation is well-established in seminal animal models (Berridge and Robinson, 2003), more work is needed to disentangle reward anticipation and consumption in humans. The current Research Topic includes 11 original articles portraying the dynamics of reward processing in humans in terms of self-report, behavioral, or neural changes.

In this topic, three articles focus on the role of emotions and personality traits in anticipatory and consummatory phases of reward processing. First, Li X. et al. asked participants to evaluate their daily experience of hedonic feelings by using experience sampling, and found that dysphoric college students reported less state anticipatory and consummatory pleasure compared with their non-dysphoric counterparts. Their results support the view that anhedonia leads to deficits in both anticipatory and consummatory phases of reward processing. Meanwhile, Huang et al. combined the classic Monetary Incentive Delay task (Knutson et al., 2000) with functional magnetic resonance imaging (fMRI) in a sample of adolescents. They discovered that callous-unemotional personality traits were positively correlated with ventral striatum activation in the anticipatory phase, but this effect was dependent on externalizing behavior. In the consummatory phase, externalizing behavior was negatively correlated with amygdala activation during punishment receipt even after controlling for callous-unemotional traits. These results help to clarify the relationship between psychopathic traits and antisocial behavior in dysfunctional reward processing. Finally, Ferreira et al. asked students to place bids to obtain food during fMRI recording and found that chronically stressed participants proposed lower bids than non-stressed ones, but there was no behavioral and neural differences during cognitive regulation of craving.

Two articles focus on the electrophysiological correlates of option evaluation, a critical stage of reward-based decision-making during which individuals evaluate and assign a value to each available option (valuation). First, Wang et al. examined event-related potential (ERP) responses of probability weight and monetary magnitude during the evaluation of a risky reward. The results showed that probability weight was encoded by the P200, the medial frontal negativity (MFN), and the late positive potential (LPP) components, whereas monetary magnitude was solely encoded by the MFN. The results demonstrated distinct temporal dynamics involved in the processing of probability weight and monetary magnitude. Meanwhile, Zhu et al. investigated both ERP and oscillatory correlates underlying the evaluation of ambiguous options using an ambiguous choice task. The authors found that delta activity was enhanced for low- vs. high-ambiguity options 200–400 ms after option onset, and for high- vs. low-reward options 400–500 ms after option onset. Ambiguity and reward information were integrated during the time window of 500–600 ms as indexed by both the P3 component and delta activity. These results help clarify neural dynamics of ambiguity vs. reward processing during option evaluation.

Four articles in this volume focus on the characteristics of reward processing in terms of its subcomponents as diverse as anticipation, learning, and consummation. Yao et al. applied an emotion (vs. sex) recognition task while participants anticipated either reward or non-reward. Their results showed that reward anticipation facilitated the processing of target information only when the target was defined by the emotional arousal of stimuli. Using a visual search task, Zhou et al. investigated the effects of prior reward learning on the processing of non-target emotional faces and found that reward history had stronger effects on fearful faces than happy faces. These two studies could further our understanding of the interaction of emotions and reward. Wu et al. investigated the impact of working memory capacity on value-driven attentional capture of reward history, and found that under the memory load condition, attentional capture of target information was more likely to be distracted by low reward-value distractors. By assessing ERPs, Yu et al. were interested in how arbitrary group membership affects the processing of reward and loss feedback in a male sample. Contrary to their expectations, the authors observed no direct support for increased in-group bias in their gambling observation task. ERP results showed that their participants employed more attentional resources during outcome processing of out-group individuals. This suggests an enhanced need for perspective taking in these cases.

Adopting a more clinically-oriented perspective, Li Q. et al. used structural equation analysis to characterize gender differences in regards to how impulsivity, coping styles, and Behavioral Inhibition/Approach System (BIS/BAS) influence internet addition in adolescents. Emotion-focused coping mediated the relationships between impulsivity/internet addition and BIS/internet addiction in girls, while problem-focused coping strategies were mostly observed to mediate the relationships between impulsivity/internet addiction and BAS/internet addiction in boys. These findings suggest that gender-sensitive training approaches should be devised to target internet addiction in adolescents more appropriately. Based on the observation that individuals undergoing evaluation of traumatic brain injury may be malingering neurocognitive deficits for compensatory benefits, Neal et al. developed a novel neural-based method for discriminating fake (i.e., simulated) from true brain injury. The authors found that individuals simulating memory deficits were characterized by delayed left frontal neural responses during recognition of studied items, which reached sensitivity of 80% and specificity of 79% in differentiating malingered from true brain injury.

In sum, the experimental findings presented in this topic shed light on the temporal dynamics (anticipation vs. consummation) of reward processing and indicate a possibility of further decomposing reward anticipation/consummation into subcomponents with distinct theoretical significance. Future research should extend existing theoretical models of reward processing by better characterizing implicated sub-processes such as stimulus-reward associations, effort computations, feedback integration, and social context effects. By addressing these issues, we may better inform more targeted prevention of and interventions for reward-related disorders.

## Author Contributions

YZ conceived the idea of the Research Topic. YZ, RG, and DP have made a substantial, direct and intellectual contribution during preparation of the editorials, and approved it for publication.

## Conflict of Interest

The authors declare that the research was conducted in the absence of any commercial or financial relationships that could be construed as a potential conflict of interest.
